# (2*E*,6*E*)-2,6-Bis(2,5-difluoro­benzyl­idene)cyclo­hexa­none

**DOI:** 10.1107/S1600536810011499

**Published:** 2010-04-02

**Authors:** Chengxi Jiang, Zhiguo Feng, Bo Song, Xiaoxia Li, Xiaokun Li

**Affiliations:** a Ministry of Education, Engineering Research Center of Bioreactor and Pharmaceutical Development, Jilin Agricultural University, Changchun 130118, People’s Republic of China; b School of Pharmacy, Wenzhou Medical College, Wenzhou, Zhejiang Province 325035, People’s Republic of China

## Abstract

In the title compound, C_20_H_14_F_4_O, a derivative of curcumin, the dihedral angle between the two aromatic rings is 27.19 (13)°. The C=C double bonds have an *E* configuration.

## Related literature

For background and related structures, see: Liang *et al.* (2007*a* ▶,*b* ▶, 2009 ▶); Zhao *et al.* (2009 ▶, 2010*a* ▶,*b* ▶).
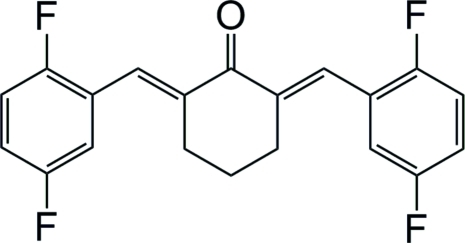


## Experimental

### 

#### Crystal data

C_20_H_14_F_4_O
*M* *_r_* = 346.31
Monoclinic, 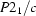

*a* = 15.824 (2) Å
*b* = 6.3128 (8) Å
*c* = 17.097 (2) Å
β = 111.756 (3)°
*V* = 1586.2 (3) Å^3^
*Z* = 4
Mo *K*α radiation
μ = 0.12 mm^−1^
*T* = 293 K
0.43 × 0.35 × 0.27 mm

#### Data collection

Bruker SMART CCD area-detector diffractometer
Absorption correction: multi-scan (*SADABS*; Bruker, 2002 ▶) *T*_min_ = 0.490, *T*_max_ = 1.000
8935 measured reflections
3425 independent reflections
2018 reflections with *I* > 2σ(*I*)
*R*_int_ = 0.049

#### Refinement

*R*[*F* ^2^ > 2σ(*F* ^2^)] = 0.056
*wR*(*F* ^2^) = 0.156
*S* = 0.93
3425 reflections
227 parameters
H-atom parameters constrained
Δρ_max_ = 0.23 e Å^−3^
Δρ_min_ = −0.24 e Å^−3^

### 

Data collection: *SMART* (Bruker, 2002 ▶); cell refinement: *SAINT* (Bruker, 2002 ▶); data reduction: *SAINT*; program(s) used to solve structure: *SHELXS97* (Sheldrick, 2008 ▶); program(s) used to refine structure: *SHELXL97* (Sheldrick, 2008 ▶); molecular graphics: *SHELXTL* (Sheldrick, 2008 ▶); software used to prepare material for publication: *SHELXTL*.

## Supplementary Material

Crystal structure: contains datablocks I, global. DOI: 10.1107/S1600536810011499/ng2751sup1.cif

Structure factors: contains datablocks I. DOI: 10.1107/S1600536810011499/ng2751Isup2.hkl

Additional supplementary materials:  crystallographic information; 3D view; checkCIF report

## Figures and Tables

**Fig. 1.**
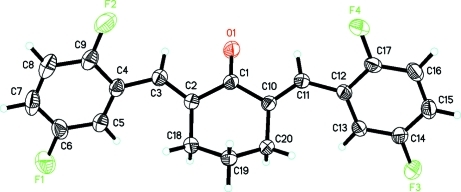
The molecular structure of the title compound, showing 30% displacement ellipsoids for the non-hydrogen atoms. Hydrogen atoms are drawn as spheres of arbitrary radius.

## supplementary crystallographic information

### Comment

The title compound, (2E,6E)-2,6-bis(2,5-difluorobenzylidene)cyclohexanone (I), is one of mono-carbonyl analogues of curcumin designed and synthesized by our group. The need for curcumin-like compounds with improved bioavailability characteristics has led to the chemical synthesis of a series of analogues, using curcumin as the primary structure. In our previous study, a series of fluorine-containing, mono-carbonyl analogues of curcumin were designed and synthesized by the deletion of β-diketone moiety, and their bioactivities were evaluated (Liang *et al.*, 2009; Zhao *et al.*, 2010a,b). Among those compounds, the cyclohexanone-containing analogues exhibited better anti-tumor properties and a wider anti-tumor spectrum than acetone- and cyclopentanone-containing analogues. Therefore, the structure of one of cyclohexanone-containing compounds (I), was further determined and analyzed using single-crystal X-ray diffraction. Accumulation of detailed structural and pharmacological data facilitated the explanation of the observed structure–activity relationships and modeling of new compounds with potential biological activity.

In this paper, we report the molecular and crystal structures of fluorine-containing, mono-carbonyl analogues of curcumin, (I). The molecule (I), consists of three ring systems, i.e., one cyclohexanone ring and two aryl rings. The central cyclohexanone ring has a distorted chair conformation, and molecular structures have an *E*-configuration towards the central olefinic bonds, exhibiting a butterfly-shaped geometry. The dihedral angle between the two terminal phenyl rings is 27.19 (13)°, and the two phenyl rings are twisted out of the plane of the central cyclohexanone on the two sides, respectively. Among these derivatives, some of them were reported of their crystal structures (Liang *et al.*, 2007a,b; Zhao *et al.*, 2009; Zhao *et al.*, 2010a,b).

### Experimental

Cyclohexanone (7.5 mmol) was dissolved in ethanol (5 ml) and crushed KOH (15 mmol) was added. The flask was immersed in a bath of crushed ice and a solution of 2,5-difluorobenzaldehyde (15 mmol) in ethanol (5 mmol) was added. The reaction mixture was stirred at 300 K and completion of the reaction was monitored by thin-layer chromatography. Ice-cold water was added to the reaction mixture after 48 h and the yellow solid that separated was filtered off, washed with water and cold ethanol, dried and purified by column chromatography on silica gel (yield: 45.3%). Single crystals of the title compound were grown in a CH_2_Cl_2_/CH_3_OH mixture (5:2 *v*/*v*) by slow evaporation (mp 132-135.4 K).

### Refinement

The H atoms were positioned geometrically (C—H = 0.93 and 0.96 Å) and refined as riding with *U*_iso_(H) = 1.2*U*_eq_(C) or 1.5*U*_eq_(methyl C).

### Crystal data

**Table:**

C_20_H_14_F_4_O	*F*(000) = 712
*M**_r_* = 346.31	*D*_x_ = 1.450 Mg m^−^^3^
Monoclinic, *P*2_1_/*c*	Mo *K*α radiation, λ = 0.71073 Å
*a* = 15.824 (2) Å	Cell parameters from 1858 reflections
*b* = 6.3128 (8) Å	θ = 2.4–22.3°
*c* = 17.097 (2) Å	µ = 0.12 mm^−^^1^
β = 111.756 (3)°	*T* = 293 K
*V* = 1586.2 (3) Å^3^	Prismatic, colorless
*Z* = 4	0.43 × 0.35 × 0.27 mm

### Data collection

**Table:**

Bruker SMART CCD area-detector diffractometer	3425 independent reflections
Radiation source: fine-focus sealed tube	2018 reflections with *I* > 2σ(*I*)
graphite	*R*_int_ = 0.049
phi and ω scans	θ_max_ = 27.0°, θ_min_ = 2.4°
Absorption correction: multi-scan (*SADABS*; Bruker, 2002)	*h* = −15→20
*T*_min_ = 0.490, *T*_max_ = 1.000	*k* = −7→8
8935 measured reflections	*l* = −21→18

### Refinement

**Table:**

Refinement on *F*^2^	Secondary atom site location: difference Fourier map
Least-squares matrix: full	Hydrogen site location: inferred from neighbouring sites
*R*[*F*^2^ > 2σ(*F*^2^)] = 0.056	H-atom parameters constrained
*wR*(*F*^2^) = 0.156	*w* = 1/[σ^2^(*F*_o_^2^) + (0.082*P*)^2^] where *P* = (*F*_o_^2^ + 2*F*_c_^2^)/3
*S* = 0.93	(Δ/σ)_max_ < 0.001
3425 reflections	Δρ_max_ = 0.23 e Å^−^^3^
227 parameters	Δρ_min_ = −0.24 e Å^−^^3^
0 restraints	Extinction correction: *SHELXL97* (Sheldrick, 2008), Fc^*^=kFc[1+0.001xFc^2^λ^3^/sin(2θ)]^-1/4^
Primary atom site location: structure-invariant direct methods	Extinction coefficient: 0.015 (2)

### Special details

**Table:**

Geometry. All esds (except the esd in the dihedral angle between two l.s. planes) are estimated using the full covariance matrix. The cell esds are taken into account individually in the estimation of esds in distances, angles and torsion angles; correlations between esds in cell parameters are only used when they are defined by crystal symmetry. An approximate (isotropic) treatment of cell esds is used for estimating esds involving l.s. planes.
Refinement. Refinement of F^2^ against ALL reflections. The weighted R-factor wR and goodness of fit S are based on F^2^, conventional R-factors R are based on F, with F set to zero for negative F^2^. The threshold expression of F^2^ > 2sigma(F^2^) is used only for calculating R-factors(gt) etc. and is not relevant to the choice of reflections for refinement. R-factors based on F^2^ are statistically about twice as large as those based on F, and R- factors based on ALL data will be even larger.

### Fractional atomic coordinates and isotropic or equivalent isotropic displacement parameters (Å^2^)

**Table:**

	*x*	*y*	*z*	*U*_iso_*/*U*_eq_	
F1	0.40753 (15)	0.4774 (3)	1.14644 (10)	0.1192 (7)	
F2	0.42662 (12)	1.2350 (2)	1.00801 (10)	0.0934 (5)	
F3	0.00929 (10)	0.5237 (2)	0.36811 (8)	0.0809 (5)	
F4	0.16575 (11)	1.2677 (2)	0.49902 (9)	0.0839 (5)	
O1	0.17234 (12)	1.1232 (3)	0.76698 (9)	0.0796 (6)	
C1	0.20606 (14)	0.9520 (4)	0.76181 (12)	0.0520 (6)	
C2	0.26784 (13)	0.8418 (3)	0.83949 (12)	0.0466 (5)	
C3	0.29758 (14)	0.9609 (4)	0.90938 (13)	0.0556 (6)	
H3	0.2766	1.0999	0.9033	0.067*	
C4	0.35876 (14)	0.9001 (4)	0.99398 (12)	0.0528 (6)	
C5	0.35519 (16)	0.7068 (4)	1.03101 (14)	0.0633 (6)	
H5	0.3125	0.6053	1.0019	0.076*	
C6	0.4149 (2)	0.6673 (5)	1.11059 (15)	0.0760 (8)	
C7	0.47922 (18)	0.8090 (6)	1.15698 (15)	0.0836 (9)	
H7	0.5196	0.7755	1.2108	0.100*	
C8	0.48225 (18)	1.0026 (5)	1.12152 (16)	0.0794 (8)	
H8	0.5247	1.1039	1.1512	0.095*	
C9	0.42230 (17)	1.0439 (4)	1.04241 (14)	0.0654 (7)	
C10	0.18662 (13)	0.8503 (3)	0.67808 (12)	0.0466 (5)	
C11	0.14905 (14)	0.9764 (3)	0.61179 (13)	0.0517 (6)	
H11	0.1403	1.1160	0.6244	0.062*	
C12	0.11951 (13)	0.9273 (3)	0.52187 (12)	0.0470 (5)	
C13	0.07950 (14)	0.7364 (3)	0.48623 (12)	0.0503 (5)	
H13	0.0731	0.6275	0.5202	0.060*	
C14	0.04988 (14)	0.7102 (4)	0.40128 (13)	0.0556 (6)	
C15	0.05733 (15)	0.8634 (4)	0.34722 (14)	0.0614 (7)	
H15	0.0367	0.8396	0.2895	0.074*	
C16	0.09649 (17)	1.0538 (4)	0.38151 (14)	0.0629 (7)	
H16	0.1025	1.1624	0.3472	0.076*	
C17	0.12608 (15)	1.0796 (3)	0.46646 (14)	0.0562 (6)	
C18	0.29633 (15)	0.6182 (3)	0.83255 (12)	0.0528 (6)	
H18A	0.2525	0.5215	0.8405	0.063*	
H18B	0.3549	0.5916	0.8769	0.063*	
C19	0.30317 (15)	0.5754 (4)	0.74764 (12)	0.0541 (6)	
H19A	0.3514	0.6616	0.7421	0.065*	
H19B	0.3188	0.4279	0.7446	0.065*	
C20	0.21500 (14)	0.6242 (3)	0.67610 (12)	0.0523 (6)	
H20A	0.2220	0.5968	0.6230	0.063*	
H20B	0.1677	0.5313	0.6796	0.063*	

### Atomic displacement parameters (Å^2^)

**Table:**

	*U*^11^	*U*^22^	*U*^33^	*U*^12^	*U*^13^	*U*^23^
F1	0.193 (2)	0.0997 (14)	0.0587 (9)	0.0260 (13)	0.0392 (11)	0.0185 (9)
F2	0.1281 (14)	0.0767 (12)	0.0776 (10)	−0.0307 (10)	0.0406 (10)	−0.0231 (8)
F3	0.0950 (11)	0.0712 (10)	0.0577 (8)	−0.0144 (8)	0.0066 (7)	−0.0117 (7)
F4	0.1145 (12)	0.0498 (9)	0.0738 (9)	−0.0116 (8)	0.0189 (9)	0.0087 (7)
O1	0.1081 (14)	0.0625 (12)	0.0539 (10)	0.0340 (10)	0.0133 (9)	−0.0087 (8)
C1	0.0591 (13)	0.0470 (14)	0.0464 (12)	0.0073 (11)	0.0155 (10)	−0.0066 (10)
C2	0.0484 (12)	0.0478 (13)	0.0427 (11)	−0.0004 (9)	0.0159 (9)	−0.0013 (9)
C3	0.0606 (14)	0.0549 (14)	0.0478 (12)	0.0038 (11)	0.0159 (11)	−0.0042 (10)
C4	0.0539 (12)	0.0624 (15)	0.0421 (11)	0.0039 (11)	0.0179 (10)	−0.0090 (10)
C5	0.0741 (16)	0.0689 (17)	0.0456 (12)	−0.0015 (13)	0.0206 (12)	−0.0073 (11)
C6	0.104 (2)	0.082 (2)	0.0416 (13)	0.0253 (16)	0.0262 (14)	0.0058 (13)
C7	0.0701 (17)	0.126 (3)	0.0422 (14)	0.0292 (18)	0.0062 (13)	−0.0182 (16)
C8	0.0680 (17)	0.109 (2)	0.0561 (16)	−0.0100 (16)	0.0176 (14)	−0.0303 (16)
C9	0.0707 (16)	0.0769 (19)	0.0497 (14)	−0.0028 (14)	0.0235 (13)	−0.0184 (13)
C10	0.0472 (11)	0.0444 (13)	0.0433 (11)	0.0023 (9)	0.0113 (9)	−0.0041 (9)
C11	0.0588 (13)	0.0431 (13)	0.0483 (12)	0.0081 (10)	0.0143 (10)	−0.0010 (9)
C12	0.0463 (11)	0.0458 (13)	0.0454 (11)	0.0090 (9)	0.0128 (9)	0.0046 (9)
C13	0.0536 (12)	0.0474 (13)	0.0445 (12)	0.0013 (10)	0.0118 (10)	0.0054 (9)
C14	0.0550 (13)	0.0537 (15)	0.0494 (13)	0.0027 (11)	0.0092 (10)	−0.0019 (11)
C15	0.0648 (15)	0.0728 (18)	0.0429 (12)	0.0142 (13)	0.0157 (11)	0.0048 (12)
C16	0.0729 (15)	0.0599 (16)	0.0547 (14)	0.0092 (13)	0.0221 (12)	0.0188 (12)
C17	0.0619 (14)	0.0417 (14)	0.0577 (14)	0.0045 (11)	0.0137 (11)	0.0063 (11)
C18	0.0606 (13)	0.0513 (14)	0.0437 (11)	0.0047 (11)	0.0161 (10)	0.0013 (10)
C19	0.0617 (13)	0.0494 (14)	0.0476 (12)	0.0110 (11)	0.0159 (10)	−0.0016 (10)
C20	0.0626 (13)	0.0462 (14)	0.0432 (11)	0.0059 (10)	0.0140 (10)	−0.0039 (9)

### Geometric parameters (Å, °)

**Table:**

F1—C6	1.371 (3)	C10—C20	1.500 (3)
F2—C9	1.355 (3)	C11—C12	1.465 (3)
F3—C14	1.360 (2)	C11—H11	0.9300
F4—C17	1.362 (2)	C12—C17	1.381 (3)
O1—C1	1.223 (2)	C12—C13	1.392 (3)
C1—C10	1.493 (3)	C13—C14	1.361 (3)
C1—C2	1.497 (3)	C13—H13	0.9300
C2—C3	1.341 (3)	C14—C15	1.372 (3)
C2—C18	1.500 (3)	C15—C16	1.380 (3)
C3—C4	1.462 (3)	C15—H15	0.9300
C3—H3	0.9300	C16—C17	1.361 (3)
C4—C9	1.380 (3)	C16—H16	0.9300
C4—C5	1.386 (3)	C18—C19	1.519 (3)
C5—C6	1.361 (3)	C18—H18A	0.9700
C5—H5	0.9300	C18—H18B	0.9700
C6—C7	1.366 (4)	C19—C20	1.508 (3)
C7—C8	1.373 (4)	C19—H19A	0.9700
C7—H7	0.9300	C19—H19B	0.9700
C8—C9	1.359 (3)	C20—H20A	0.9700
C8—H8	0.9300	C20—H20B	0.9700
C10—C11	1.331 (3)		
			
O1—C1—C10	120.60 (18)	C13—C12—C11	124.00 (19)
O1—C1—C2	120.36 (18)	C14—C13—C12	119.5 (2)
C10—C1—C2	119.03 (19)	C14—C13—H13	120.2
C3—C2—C1	115.35 (19)	C12—C13—H13	120.2
C3—C2—C18	125.57 (18)	F3—C14—C13	118.2 (2)
C1—C2—C18	118.93 (17)	F3—C14—C15	118.3 (2)
C2—C3—C4	128.3 (2)	C13—C14—C15	123.5 (2)
C2—C3—H3	115.9	C14—C15—C16	117.7 (2)
C4—C3—H3	115.9	C14—C15—H15	121.2
C9—C4—C5	116.7 (2)	C16—C15—H15	121.2
C9—C4—C3	119.3 (2)	C17—C16—C15	118.7 (2)
C5—C4—C3	124.0 (2)	C17—C16—H16	120.6
C6—C5—C4	119.1 (2)	C15—C16—H16	120.6
C6—C5—H5	120.4	C16—C17—F4	117.7 (2)
C4—C5—H5	120.4	C16—C17—C12	124.4 (2)
C5—C6—C7	123.5 (3)	F4—C17—C12	117.9 (2)
C5—C6—F1	117.7 (3)	C2—C18—C19	111.88 (17)
C7—C6—F1	118.8 (2)	C2—C18—H18A	109.2
C6—C7—C8	117.8 (2)	C19—C18—H18A	109.2
C6—C7—H7	121.1	C2—C18—H18B	109.2
C8—C7—H7	121.1	C19—C18—H18B	109.2
C9—C8—C7	119.0 (3)	H18A—C18—H18B	107.9
C9—C8—H8	120.5	C20—C19—C18	111.50 (18)
C7—C8—H8	120.5	C20—C19—H19A	109.3
F2—C9—C8	118.3 (2)	C18—C19—H19A	109.3
F2—C9—C4	117.9 (2)	C20—C19—H19B	109.3
C8—C9—C4	123.8 (3)	C18—C19—H19B	109.3
C11—C10—C1	115.29 (19)	H19A—C19—H19B	108.0
C11—C10—C20	126.35 (18)	C10—C20—C19	111.77 (17)
C1—C10—C20	118.28 (17)	C10—C20—H20A	109.3
C10—C11—C12	129.4 (2)	C19—C20—H20A	109.3
C10—C11—H11	115.3	C10—C20—H20B	109.3
C12—C11—H11	115.3	C19—C20—H20B	109.3
C17—C12—C13	116.12 (19)	H20A—C20—H20B	107.9
C17—C12—C11	119.8 (2)		
			
O1—C1—C2—C3	−13.6 (3)	C2—C1—C10—C20	11.5 (3)
C10—C1—C2—C3	166.2 (2)	C1—C10—C11—C12	−178.3 (2)
O1—C1—C2—C18	170.6 (2)	C20—C10—C11—C12	5.2 (4)
C10—C1—C2—C18	−9.7 (3)	C10—C11—C12—C17	−146.9 (2)
C1—C2—C3—C4	−179.3 (2)	C10—C11—C12—C13	36.8 (4)
C18—C2—C3—C4	−3.8 (4)	C17—C12—C13—C14	0.1 (3)
C2—C3—C4—C9	141.8 (2)	C11—C12—C13—C14	176.5 (2)
C2—C3—C4—C5	−40.8 (4)	C12—C13—C14—F3	−178.60 (18)
C9—C4—C5—C6	−1.8 (3)	C12—C13—C14—C15	0.2 (3)
C3—C4—C5—C6	−179.3 (2)	F3—C14—C15—C16	178.34 (19)
C4—C5—C6—C7	0.1 (4)	C13—C14—C15—C16	−0.5 (4)
C4—C5—C6—F1	177.8 (2)	C14—C15—C16—C17	0.5 (4)
C5—C6—C7—C8	1.1 (4)	C15—C16—C17—F4	179.0 (2)
F1—C6—C7—C8	−176.6 (2)	C15—C16—C17—C12	−0.2 (4)
C6—C7—C8—C9	−0.5 (4)	C13—C12—C17—C16	0.0 (3)
C7—C8—C9—F2	−179.5 (2)	C11—C12—C17—C16	−176.6 (2)
C7—C8—C9—C4	−1.4 (4)	C13—C12—C17—F4	−179.30 (18)
C5—C4—C9—F2	−179.4 (2)	C11—C12—C17—F4	4.1 (3)
C3—C4—C9—F2	−1.7 (3)	C3—C2—C18—C19	−143.7 (2)
C5—C4—C9—C8	2.5 (4)	C1—C2—C18—C19	31.7 (3)
C3—C4—C9—C8	−179.8 (2)	C2—C18—C19—C20	−56.2 (3)
O1—C1—C10—C11	14.5 (3)	C11—C10—C20—C19	140.9 (2)
C2—C1—C10—C11	−165.28 (19)	C1—C10—C20—C19	−35.6 (3)
O1—C1—C10—C20	−168.7 (2)	C18—C19—C20—C10	58.3 (3)

### Hydrogen-bond geometry (Å, °)

**Table:**

*D*—H···*A*	*D*—H	H···*A*	*D*···*A*	*D*—H···*A*
C16—H16···O1^i^	0.93	2.46	3.343 (3)	158

Symmetry codes: (i) *x*, −*y*+5/2, *z*−1/2.
